# Supplementary material for: Third molar agenesis in modern humans with and without agenesis of other teeth
Source: PeerJ. 2020 Nov 17;8:e10367. doi: 10.7717/peerj.10367 (PMC7678444; doi:10.7717/peerj.10367)
Supplement: Supplemental Information 1 [file peerj-08-10367-s001.docx]

**Supplemental Table S1**. Distribution of the total number of missing teeth per individual in the agenesis sample, excluding third molars.

|  | Number of missing teeth | Frequency | Percentage (%) |
| --- | --- | --- | --- |
|  | 1 | 117 | 38.6 |
|  | 2 | 101 | 33.3 |
|  | 3 | 24 | 7.9 |
|  | 4 | 24 | 7.9 |
|  | 5 | 10 | 3.3 |
|  | 6 | 3 | 1 |
|  | 7 | 6 | 2 |
|  | 8 | 2 | 0.7 |
|  | 9 | 4 | 1.3 |
|  | 10 | 4 | 1.3 |
|  | 11 | 1 | 0.3 |
|  | 12 | 2 | 0.7 |
|  | 13 | 1 | 0.3 |
|  | 14 | 3 | 1 |
|  | 20 | 1 | 0.3 |
| Total | 799 | 303 | 100 |
