# Supplementary material for: Third molar agenesis in modern humans with and without agenesis of other teeth
Source: PeerJ. 2020 Nov 17;8:e10367. doi: 10.7717/peerj.10367 (PMC7678444; doi:10.7717/peerj.10367)
Supplement: Supplemental Information 2 [file peerj-08-10367-s002.docx]

**Supplemental Table S2.** Most common patterns of tooth agenesis observed in the agenesis group including third molars.

|  | Frequency (%) | Missing teeth |  | Frequency (%) | Missing teeth |
| --- | --- | --- | --- | --- | --- |
| **Maxilla** | | | **Mandible** | | |
| 1 | 31/220 (14.1) | 12, 22 | 1 | 30/234 (12.8) | 35, 45 |
| 2 | 30/220 (13.6) | 18, 28 | 2 | 24/234 (10.3) | 35 |
| 3 | 17/220 (7.7) | 22 | 3 | 20/234 (8.5) | 45  or  35, 38, 45, 48 |
| 4 | 16/220 (7.3) | 12 | 4 | 16/234 (6.8) | 38, 48 |
| 5 | 12/220 (5.5) | 15, 18, 25, 28 | 5 | 13/234 (5.6) | 38, 45, 48 |
| Overall | 106/220 (48.2) |  | Overall | 103/234 (44.0) |  |
| **Whole dentition** | | | | | |
| 1 | 22/303 (7.3) | 12, 22 | | | |
| 2 | 16/303 (5.3) | 35 or 45 | | | |
| 3 | 15/303 (5.0) | 22 | | | |
| 4 | 14/303 (4.6) | 35, 45 | | | |
| 5 | 12/303 (4.0) | 12 | | | |
| Overall | 79/303 (26.1) |  | | | |
