# Supplementary material for: Third molar agenesis in modern humans with and without agenesis of other teeth
Source: PeerJ. 2020 Nov 17;8:e10367. doi: 10.7717/peerj.10367 (PMC7678444; doi:10.7717/peerj.10367)
Supplement: Supplemental Information 3 [file peerj-08-10367-s003.docx]

**Supplemental Table S3.** Most common patterns of third molar agenesis observed in control individuals.

|  | Frequency (%) | Missing teeth |  | Frequency (%) | Missing teeth |
| --- | --- | --- | --- | --- | --- |
| **Maxilla** | | | **Mandible** | | |
| 1 | 26/43 (60.5) | 18, 28 | 1 | 27/48 (56.3) | 38, 48 |
| 2 | 11/43 (25.6) | 18 | 2 | 12/48 (25) | 48 |
| 3 | 6/43 (14.0) | 28 | 3 | 9/48 (18.8) | 38 |
| Overall | 43/43 (100) |  | Overall | 48/48 (100) |  |
| **Whole dentition** | | | | | |
| 1 | 17/62 (27.4) | 18, 28, 38, 48 | | | |
| 2 | 9/62 (14.5) | 38, 48 | | | |
| 3 | 6/62 (9.7) | 38 | | | |
| 4 | 5/62 (8.1) | 28 or 18  or 18, 48 | | | |
| 5 | 4/62 (6.5) | 18, 28 or  48 | | | |
| Overall | 50/62 (80.6) |  | | | |
