# Supplementary material for: Third molar agenesis in modern humans with and without agenesis of other teeth
Source: PeerJ. 2020 Nov 17;8:e10367. doi: 10.7717/peerj.10367 (PMC7678444; doi:10.7717/peerj.10367)
Supplement: Supplemental Information 4 [file peerj-08-10367-s004.docx]

**Supplemental Table S4.** Most common patterns of third molar agenesis observed in the agenesis subjects.

|  | Frequency (%) | Missing teeth |  | Frequency (%) | Missing teeth |
| --- | --- | --- | --- | --- | --- |
| **Maxilla** | | | **Mandible** | | |
| 1 | 88/118 (74.6) | 18, 28 | 1 | 88/124 (71.0) | 38, 48 |
| 2 | 17/118 (14.4) | 28 | 2 | 20/124 (16.1) | 38 |
| 3 | 13/118 (11) | 18 | 3 | 16/124 (12.9) | 48 |
| Overall | 118/118 (100) |  | Overall | 124/124 (100) |  |
| **Whole dentition** | | | | | |
| 1 | 59/154 (38.3) | 18, 28, 38, 48 | | | |
| 2 | 19/154 (12.3) | 38, 48 | | | |
| 3 | 16/154 (10.4) | 18, 28 | | | |
| 4 | 13/154 (8.4) | 38 | | | |
| 5 | 8/154 (5.2) | 28 or 18, 28, 48 | | | |
| Overall | 123/154 (79.9) |  | | | |
