# Supplementary figures and images for: Third molar agenesis in modern humans with and without agenesis of other teeth
Source: PeerJ. 2020 Nov 17;8:e10367. doi: 10.7717/peerj.10367 (PMC7678444; doi:10.7717/peerj.10367)

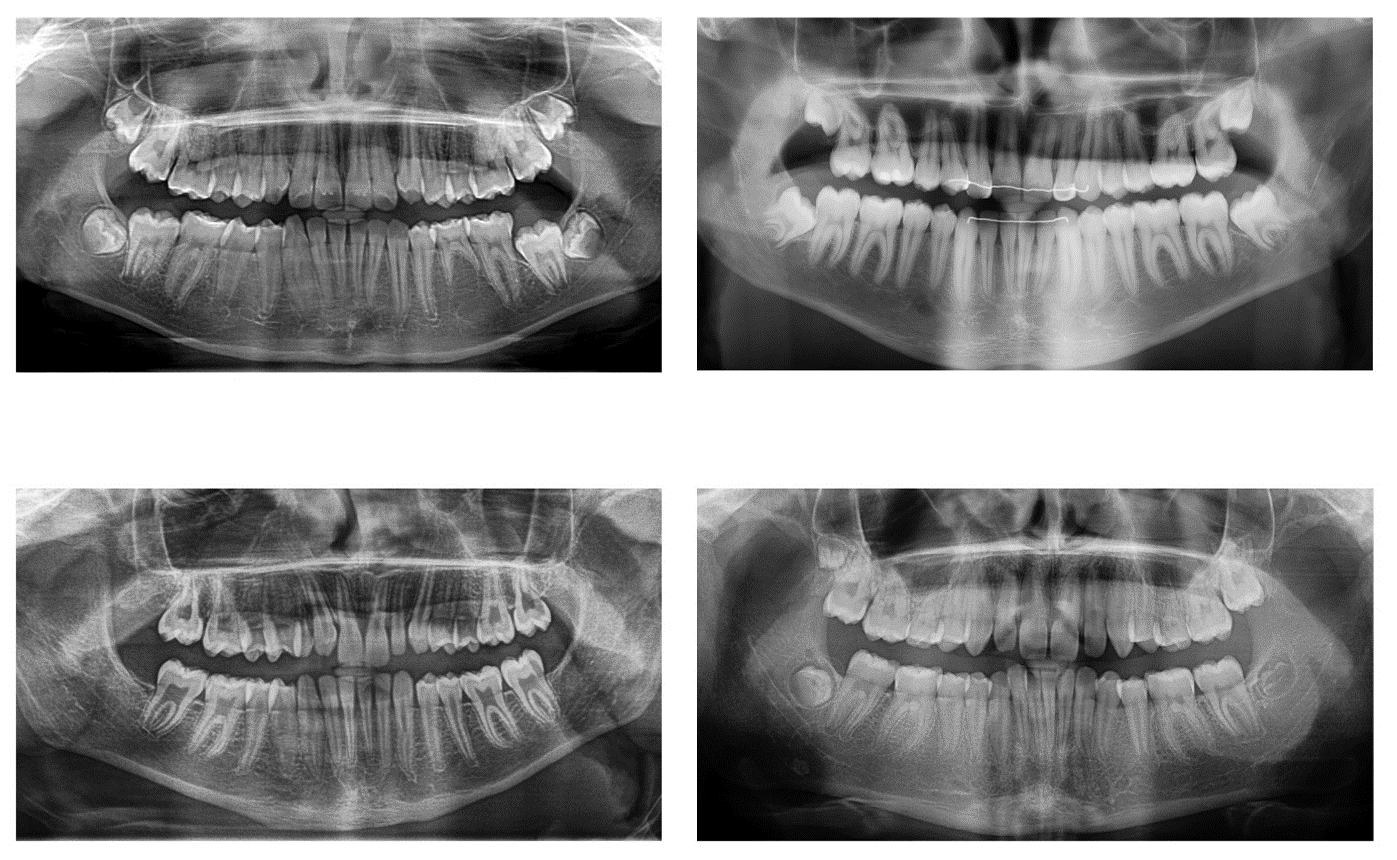

Supplement: Supplemental Information 5 [file peerj-08-10367-s005.png]
